# In Memoriam—Dr. Guy A. T. Lincoln

**Published:** 1895-12

**Authors:** 


### IN MEMORIAM—DR. GUY A. T. LINCOLN.

It is with regret and mortification that the Editor finds that a capital mistake was made in the name of the late Dr. Lincoln, in the obituary notice of him in the November number, at page 506. The corrected name appears at the head of this article. It may also be remarked that the second member of the committee was Dr. R. L. Thurston, and not Thornton, as printed.

